# Pairwise Agreement Among Field-Based Body-Fat Estimation Methods in Physically Active Adults: A Cross-Sectional Method-Comparison Study

**DOI:** 10.3390/jfmk11040401

**Published:** 2026-10-02

**Authors:** Raynier Zambrano-Villacres, Kathy Elisabeth Verdugo Verdugo, Washington David Guevara Castillo, Nelly Andrade Mejia, Jaen Cagua-Ordoñez, Daniel Simancas-Racines

**Affiliations:** 1 Facultad de Ciencias de la Salud y Desarrollo Humano, Universidad ECOTEC, Km.13.5 Samborondón, Samborondón EC092302, Ecuador; razambrano@ecotec.edu.ec; 2 Escuela de Posgrado, Doctorado en Ciencias de la Salud, Instituto Universitario Italiano de Rosario (IUNIR), Rosario S2000CTT, Argentina; 3 Escuela de Nutriología, Facultad de Ciencias Médicas, de la Salud y la Vida, Universidad Internacional del Ecuador, Quito 170411, Ecuador; 4 Facultad de Ciencias de la Salud, Universidad Espíritu Santo, Samborondón 0901952, Ecuador; 5 Center for Evidence Ecosystems, Implementation Science, and Decision-Making (CIDES), Facultad de Ciencias de la Salud y Bienestar Humano, Universidad Tecnológica Indoamérica, Ambato 180150, Ecuador

**Keywords:** body composition, body fat, anthropometry, bioelectrical impedance analysis, method comparison, limits of agreement, physical activity, health promotion

## Abstract

**Background**: Field-based body-fat estimates may differ substantially across methods, but similarity in group averages does not necessarily indicate close individual-level agreement. This study characterized pairwise numerical agreement among four field-based body-fat estimation methods without designating a criterion reference. **Methods**: Forty physically active adults (19 women and 21 men) underwent same-session assessment using archived Carter-derived and Faulkner-derived anthropometric estimates, single-frequency bioelectrical impedance analysis (BIA; Omron HBF-514C), and multifrequency BIA (InBody 120). Agreement was evaluated using paired differences, 95% limits of agreement (LoA), bootstrap confidence intervals, and analyses of non-constant mean and variance structures. Difference-on-paired-mean patterns were additionally examined considering mathematical coupling and unequal method variances. **Results**: Carter-derived estimates averaged 4.16 percentage points (pp) lower than Faulkner-derived estimates, whereas Omron estimates averaged 5.34 pp higher than InBody estimates. Carter–Faulkner showed an approximately stable mean difference but increasing dispersion across the measurement range, supporting level-specific LoA. Faulkner–InBody showed a small mean difference (−1.48 pp) despite wide global LoA (−12.91 to 9.94 pp). Anthropometry–BIA comparisons showed negative difference-on-paired-mean slopes (−0.58 to −0.72); however, these patterns were algebraically consistent with mathematical coupling and unequal method variances and were not interpreted as independent evidence of adiposity-level effects. **Conclusions**: The methods showed systematic pairwise differences and substantial individual-level disagreement. Because no criterion method, replicate measurements, or prespecified application-specific acceptability threshold was available, the findings establish neither accuracy, superiority, nor interchangeability among the methods.

## 1. Introduction

Body-composition assessment is widely used in sport, exercise, and health settings to characterize adiposity and support nutritional, training, and clinical decision-making [1,2]. In field settings, however, body fat is generally estimated indirectly rather than measured directly. The resulting estimates depend on the underlying measurement model, prediction equation, device characteristics, assessment conditions, and characteristics of the population in which the method or prediction model was developed or validated [3,4]. Consequently, methods reporting body fat in the same percentage units should not be assumed to provide numerically equivalent estimates.

Anthropometry and bioelectrical impedance analysis (BIA) are among the most accessible approaches for field-based body-composition assessment [2,5]. Anthropometric approaches estimate body composition from skinfold thicknesses and other body measurements through prediction equations whose performance depends on model specification and the population in which they were developed or validated [6,7]. Accordingly, different anthropometric equations may yield different body-composition estimates even when applied to the same individual [6].

BIA estimates body composition from electrical measurements combined with anthropometric and demographic information through device- or equation-specific prediction models [3,5]. Commercial BIA systems may differ in electrical frequency, electrode configuration, body pathway, measurement technology, and prediction procedures [8,9]. Hydration, fluid distribution, recent exercise, food and fluid intake, and other pre-assessment conditions may additionally influence impedance measurements or derived body-composition estimates [5,10]. Thus, numerical disagreement may occur not only between anthropometry and BIA but also between specific equations or devices that belong to the same broad methodological category [6,8].

Evaluating such differences requires distinguishing agreement from association and accuracy. Strong correlation between two methods does not imply sufficiently close agreement for measurements obtained in the same individual, and similarity in group means can coexist with substantial individual-level disagreement [11,12]. Bland–Altman analysis addresses agreement by examining paired differences and their limits of agreement (LoA), thereby describing the magnitude and dispersion of differences between two measurement methods [11,12]. Interpretation of the LoA should consider both their uncertainty and whether the observed disagreement is acceptable for the intended application [11].

Patterns of paired differences across the measurement range may provide additional descriptive information, particularly when either the mean difference or its variability is non-constant [11,13]. However, such patterns require careful interpretation. Because the paired difference and paired mean are constructed from the same measurements, their relationship may be influenced by the variance structure of the compared methods [13,14]. Consequently, an association between the difference and paired mean should not, by itself, be interpreted as evidence that disagreement changes with the underlying true value.

These considerations are particularly relevant in physically active populations, in whom body-composition estimates may inform nutritional assessment, training decisions, participant characterization, and longitudinal monitoring [1,2]. Method-specific numerical differences may therefore have practical implications when measurements obtained using different equations or devices are compared. At the same time, agreement observed for a particular pair should not be generalized to all anthropometric equations or BIA devices because individual methods incorporate specific measurement procedures, prediction models, and assumptions [6,8].

Accordingly, the aim of this study was to characterize pairwise numerical agreement among four field-based body-fat estimation methods in physically active adults: archived Carter-derived and Faulkner-derived anthropometric estimates, the single-frequency Omron HBF-514C, and the multifrequency InBody 120. All six pairwise comparisons were evaluated without designating any method as a criterion reference. The primary focus was the magnitude and uncertainty of paired differences and LoA, including assessment of non-constant dispersion where appropriate. Patterns of differences across paired means were examined descriptively and interpreted considering mathematical coupling and unequal method variances. The study was designed to characterize comparative numerical agreement rather than determine accuracy, superiority, or interchangeability.

## 2. Materials and Methods

### 2.1. Study Design and Participants

This cross-sectional method-comparison study evaluated pairwise numerical agreement among four field-based methods used to estimate body-fat percentage in physically active adults. Data were collected in Quito, Ecuador, at approximately 2850 m above sea level, between April and May 2025. All four assessments were performed in each participant during a single study session to minimize between-session biological variation.

The study was designed to compare the numerical estimates produced by the methods rather than to assess their diagnostic or criterion validity. No criterion or multicomponent reference method was included; therefore, none of the four methods was designated as a reference standard. The reporting and interpretation of agreement analyses followed established recommendations for method-comparison studies [12,15].

Forty physically active adults (19 women and 21 men) participated in the study. Eligibility criteria included adulthood (≥18 years) and regular participation in physical activity or exercise; no upper age limit was specified. Participants with conditions or circumstances that precluded safe or technically appropriate anthropometric or bioelectrical impedance analysis (BIA) assessment were excluded according to the original study protocol.

### 2.2. Assessment Setting and Measurement Standardization

All measurements were obtained during the same session under standardized pre-assessment instructions. Participants were instructed to avoid strenuous exercise for at least 12 h, fast for at least 4 h, avoid alcohol and tobacco for 48 h, maintain their usual hydration while avoiding excessive fluid intake immediately before testing, remove metallic objects, and empty their bladder approximately 30 min before assessment. These instructions describe the preparation protocol used in the study; adherence to the instructions and hydration status were not objectively verified.

Menstrual-cycle phase was not systematically recorded. Individual duration of residence or acclimatization at the altitude of the testing site was also not systematically recorded and therefore could not be incorporated into the analyses. These factors were considered when interpreting the findings rather than being treated as controlled experimental conditions.

### 2.3. Anthropometric Assessment

Anthropometric measurements were performed by an International Society for the Advancement of Kinanthropometry (ISAK) Level 1 anthropometrist under the supervision of an ISAK Level 3 instructor, using standardized anatomical landmarks and procedures [16,17]. Measurements were obtained on the right side of the body. Technical error of measurement remained below 5% for skinfold measurements and below 1% for non-skinfold anthropometric measurements.

Body mass was recorded in kilograms (kg), stature in centimeters (cm), and skinfold thickness in millimeters (mm). Eight skinfold sites were assessed: triceps, subscapular, biceps, iliac crest, supraspinale, abdominal, front thigh, and medial calf. Skinfold thickness was measured using a Cescorf Innovare skinfold caliper, and stature was measured using a Seca 213 stadiometer. Anthropometric equipment was checked and calibrated before data collection.

The original analytical dataset contained two archived body-fat percentage variables labelled as Carter and Faulkner estimates. These two estimates were included in the present method-comparison study because they were the anthropometry-derived body-fat variables available in the original analytical dataset; they were not retrospectively selected from among alternative anthropometric equations for the present analysis. The complete computational workflow used during the original assessment, including the exact equation implementation and intermediate calculations from the raw anthropometric measurements, was not retained in the analytical dataset available for the present method-comparison analysis. Consequently, these values were not retrospectively reconstructed or recalculated because doing so could not establish that a reconstructed implementation was identical to that originally used. They were therefore analyzed as archived method-specific outputs and are referred to throughout this study as Carter-derived and Faulkner-derived estimates.

Published anthropometric approaches historically associated with the Carter and Faulkner labels, together with their development and attribution contexts, have been described in the literature [16,17]. These references provide methodological and historical context for the labels retained in the original dataset but should not be interpreted as retrospective verification of the exact computational implementation used to generate the archived estimates analyzed in this study. Accordingly, the present analysis evaluates the numerical behavior of the archived estimates and does not constitute a revalidation or recalibration of the underlying anthropometric equations.

### 2.4. Bioelectrical Impedance Assessment

Single-frequency BIA was performed using the Omron HBF-514C, a hand-to-foot device operating at 50 kHz. Participants stood barefoot on the foot electrodes and held the hand electrodes in the manufacturer’s recommended position. The body-fat percentage reported by the device was recorded directly.

Multifrequency BIA was performed using the InBody 120, which uses an eight-point tactile electrode system and electrical frequencies of 20 and 100 kHz. Participants stood barefoot on the platform, maintained contact with the hand electrodes, and remained in the standardized measurement position during assessment. Device-reported body-fat percentage was recorded directly.

Both devices generate body-composition estimates using manufacturer-specific prediction procedures. These algorithms were used without modification, and device-generated estimates were treated as method-specific outputs rather than direct measurements of body fat. Neither BIA device, including the InBody 120, was considered a criterion or gold-standard method.

### 2.5. Outcome Definition and Pairwise Comparison Framework

The primary analytical outcome was body-fat percentage. Four method-specific estimates were evaluated: archived Carter-derived anthropometry, archived Faulkner-derived anthropometry, single-frequency BIA using the Omron HBF-514C, and multifrequency BIA using the InBody 120. Because no criterion or multicomponent reference method was included, none of the four methods was designated as a reference standard.

All six possible pairwise comparisons were evaluated: Carter–Faulkner, Carter–Omron, Carter–InBody, Faulkner–Omron, Faulkner–InBody, and Omron–InBody. For methods *A* and *B*, the participant-specific paired difference was defined as

*D_i_* = *A_i_* − *B_i_*,

and the corresponding paired mean as

$$M_{i}=\frac{A_{i}+B_{i}}{2}.$$

Thus, a negative difference indicates that the first-listed method produced a numerically lower estimate than the second-listed method, whereas a positive difference indicates a higher estimate.

The primary agreement estimands were the mean paired difference and the 95% limits of agreement (LoA), together with their uncertainty. Participant-level absolute discrepancy,

|*D_i_*| = |*A_i_* − *B_i_*|,

was examined as a complementary measure of the magnitude of individual disagreement. No post hoc threshold was used to classify agreement as clinically or practically acceptable and no pair was classified as equivalent or interchangeable because an application-specific acceptable difference had not been prespecified.

Agreement observed for an individual pair was interpreted specifically for the methods compared and was not extrapolated to anthropometric methods or BIA devices as general methodological categories.

### 2.6. Statistical Analysis

Analyses were performed in R version 4.6.1 (R Foundation for Statistical Computing, Vienna, Austria). Continuous participant characteristics and method-specific body-fat estimates were summarized using means and standard deviations (SD), together with ranges where informative; categorical variables were summarized using frequencies and percentages. The analytical dataset was audited for missingness before pairwise analyses. For each comparison, analyses were based on participants with non-missing values for both methods, and the corresponding pairwise sample size was reported. No imputation of missing body-fat estimates was performed.

#### 2.6.1. Primary Agreement Analysis

For each method pair, the mean paired difference was calculated as

$$\underline{D}=\frac{1}{n}\sum_{i=1}^{n}D_{i},$$

and conventional 95% LoA were initially calculated as

$$\underline{D}\pm 1.96s_{D},$$

where *s_D_* is the SD of the paired differences [11,12]. Uncertainty around agreement estimates was quantified using participant-level nonparametric bootstrap resampling with 10,000 replicates, preserving the paired structure of the data.

The mean difference was interpreted as the average numerical offset between the two methods rather than as measurement bias relative to truth, because neither method constituted a criterion reference. Likewise, LoA were interpreted as describing the distribution of individual between-method differences in the study population rather than as evidence of clinical or practical acceptability. Because no acceptable disagreement margin was defined *a priori*, the magnitude of the LoA was described but not formally classified as acceptable or unacceptable.

#### 2.6.2. Assessment of Non-Constant Dispersion

The assumption of approximately constant variability of paired differences across the measurement range was examined for each comparison. Constant-variance models were compared with generalized least-squares models, allowing residual variability to change with the paired mean using power (varPower) and exponential (varExp) variance functions. Models were fitted using maximum likelihood for comparison by Akaike information criterion (AIC).

A reduction of ≥4 AIC units relative to the constant-variance model was used as the model-selection criterion. When this criterion was met, agreement was primarily characterized using level-specific LoA derived from the selected heteroscedastic model, while conventional constant-SD LoA were retained as a global descriptive summary. For the selected heteroscedastic model, uncertainty in level-specific estimates was quantified using a participant-level percentile bootstrap with 10,000 resamples, refitting the model in each resample.

This approach allowed the mean structure and variance structure of paired differences to be considered separately. Consequently, an approximately constant mean difference was not assumed to imply constant LoA when the dispersion of the differences changed across the measurement range.

#### 2.6.3. Difference-on-Paired-Mean Patterns and Mathematical Coupling

Linear regressions of the paired difference *D_i_* on the paired mean *M_i_* were examined to describe the observed geometry of between-method differences across the measurement range. However, these regressions were not interpreted as independent tests whose disagreement changes with the underlying level of body fat.

This restriction was necessary because the paired difference and paired mean are mathematically coupled. Specifically,

$$\mathrm{Cov}\left(A,-B\frac{A+B}{2}\right)=\frac{\mathrm{Var}\left(A\right)-\mathrm{Var}\left(B\right)}{2}.$$

Therefore, a non-zero difference-on-paired-mean slope can arise from unequal marginal variances of the two methods, even in the absence of an independently identifiable biological or measurement-level effect [14]. For each method pair, we consequently quantified the variances and SDs of both methods, their variance ratio, the observed covariance between the paired difference and paired mean, the covariance implied by the algebraic identity above, and the corresponding observed and algebraically implied regression slopes. Participant-level bootstrap resampling was used to characterize uncertainty in these quantities.

Accordingly, difference-on-paired-mean slopes and conditional mean differences were retained as descriptive summaries of the observed paired data but were not interpreted as demonstrating genuine proportional bias or an independent body-fat-level effect.

#### 2.6.4. Measurement-Error Sensitivity Analysis

Because both methods in each comparison are subject to measurement error, ordinary least-squares regression of one method on another cannot identify a unique structural relationship without assumptions concerning their relative error variances. As a sensitivity analysis, Deming regressions were therefore evaluated across assumed error-variance ratios of

*λ* = 0.25, 0.5, 1, 2, *and* 4

These analyses were used only to examine how assumptions regarding relative measurement error affected the estimated method-to-method relationship. Because replicate measurements, external estimates of method-specific measurement-error variance, and a criterion method were unavailable, *λ* could not be empirically identified from the present data. Deming regression was therefore treated as an assumption-based sensitivity analysis and not as evidence that a particular proportional-difference model was correct.

#### 2.6.5. Secondary and Sensitivity Analyses

Absolute participant-level differences were summarized to complement signed agreement estimates. Intraclass correlation coefficients were calculated as secondary measures of consistency but were not used as substitutes for Bland–Altman agreement because high consistency may coexist with systematic numerical differences or wide LoA.

Exploratory sex-stratified Bland–Altman summaries were calculated separately for women and men. For the four anthropometry–BIA comparisons showing negative descriptive difference-on-paired-mean slopes, additional models adjusted for sex and age were fitted to examine how adjustment for these participant characteristics affected the descriptive paired-mean association. Because the sex-specific samples were small, sex was unevenly distributed across sport categories, and adjustment does not remove the mathematical coupling between paired differences and paired means, these analyses were considered exploratory and were not interpreted as evidence of sex-specific method effects, effect modification, or an independent body-fat-level effect.

Influence diagnostics, including Cook’s distance and leave-one-participant-out analyses, were used to assess whether principal regression patterns were disproportionately determined by individual observations. Additional precision analyses quantified uncertainty in the estimated LoA and explored the sample sizes that would be required to obtain narrower LoA confidence intervals in future studies.

All inferential estimates were accompanied by 95% confidence intervals where appropriate. Statistical analyses were interpreted primarily according to effect magnitude, uncertainty, agreement structure, and model diagnostics rather than solely according to null-hypothesis significance testing.

### 2.7. Ethical Considerations

The study was conducted in accordance with the Declaration of Helsinki and was approved by the Bioethics Committee of the Universidad Internacional del Ecuador (protocol code OB_04_2025_UIDE_DG, version 1; approval date: 17 March 2025). All participants received information regarding the study objectives and procedures and provided written informed consent before enrollment. Participation was voluntary, and the analytical database was anonymized before statistical analysis.

## 3. Results

### 3.1. Participant Characteristics and Distribution of Body-Fat Estimates

All 40 participants had complete body-fat estimates for the four primary assessment methods and were included in all pairwise analyses. The sample comprised 19 women (47.5%) and 21 men (52.5%), with a mean age of 34.8 years (SD 8.8; range 18–60 years). Participants represented seven physical-activity or sport groups, most commonly athletics (n = 14), mountaineering (n = 10), and CrossFit (n = 6). Participant characteristics are summarized in Table 1.

**Figure 1:**
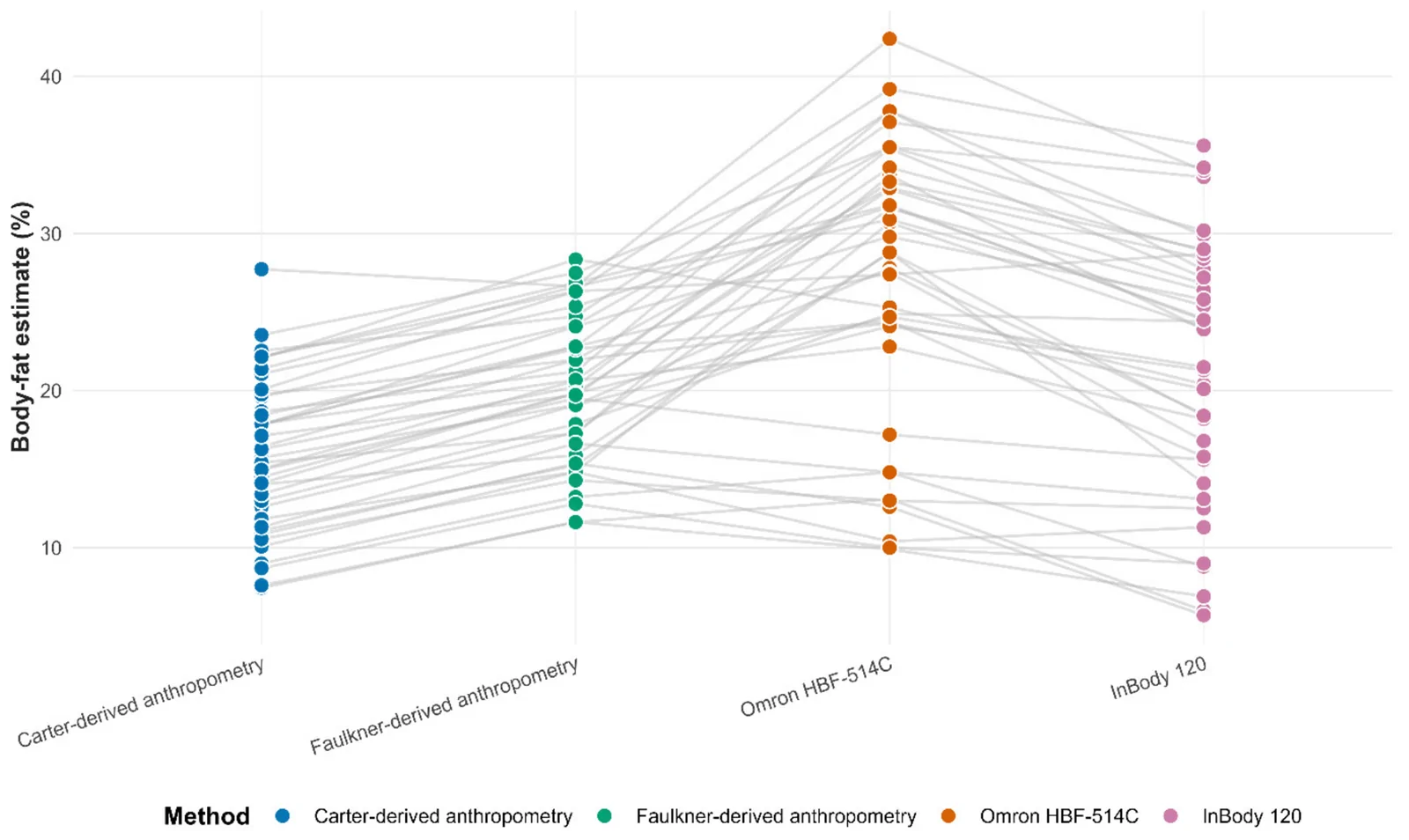
Individual body-fat estimates across assessment methods. Individual body-fat percentage estimates obtained using Carter-derived anthropometry, Faulkner-derived anthropometry, the Omron HBF-514C, and the InBody 120. Grey lines connect measurements from the same participant, and colored points identify the assessment method. The figure illustrates participant-level variability and systematic numerical differences across methods. No method was considered a criterion or gold-standard measure.

Body-fat estimates differed across the four methods. Carter-derived anthropometry produced the lowest mean estimate (16.04%, SD 4.85), followed by Faulkner-derived anthropometry (20.20%, SD 4.74), InBody 120 (21.68%, SD 8.27), and Omron HBF-514C (27.02%, SD 8.99). The corresponding observed ranges were 7.45–27.73%, 11.63–28.35%, 5.70–35.60%, and 9.90–42.40%, respectively. The two BIA devices showed greater between-participant dispersion than the two archived anthropometry-derived estimates. Complete descriptive distributions for the four methods are provided in Supplementary Table S1. Individual participant estimates across methods are shown in Figure 1.

### 3.2. Pairwise Differences and Global Limits of Agreement

Pairwise comparisons showed systematic numerical differences of varying magnitude (Table 2). Carter-derived estimates were, on average, 4.16 percentage points (pp) lower than Faulkner-derived estimates (bootstrap 95% CI, −4.59 to −3.68), with classical 95% LoA of −7.04 to −1.27 pp. Omron estimates were, on average, 5.34 pp higher than InBody estimates (95% CI, 4.28 to 6.42), with classical LoA of −1.46 to 12.13 pp.

Comparisons between the individual anthropometric and BIA methods generally showed greater participant-level dispersion. Mean differences were −10.98 pp for Carter–Omron, −5.64 pp for Carter–InBody, −6.82 pp for Faulkner–Omron, and −1.48 pp for Faulkner–InBody. Their corresponding classical LoA were −22.67 to 0.72 pp, −16.32 to 5.04 pp, −19.93 to 6.29 pp, and −12.91 to 9.94 pp, respectively. Thus, a relatively modest overall mean difference did not necessarily imply close participant-level agreement. This was particularly apparent for Faulkner–InBody, for which the mean difference was −1.48 pp, whereas the classical LoA spanned approximately 23 pp.

Classical LoA provide global summaries across the observed measurement range. Because residual dispersion was not constant for one comparison, level-specific agreement estimates are presented in Section 3.3. A graphical summary of mean pairwise differences and classical LoA is provided in Supplementary Figure S1, while conventional Bland–Altman plots for all six pairwise comparisons are presented in Supplementary Figure S3.

### 3.3. Non-Constant Dispersion and Level-Specific Limits of Agreement

Assessment of the residual variance structure showed evidence of non-constant dispersion for the Carter–Faulkner comparison. A varPower variance model provided a substantially better fit than the corresponding constant-variance model (AIC 136.29 vs. 149.15; ΔAIC = −12.86). None of the other five pairwise comparisons met the ≥4-AIC-unit criterion used to favor a non-constant variance structure (Supplementary Table S2).

For Carter–Faulkner, the fitted mean difference remained approximately stable across the evaluated paired-mean range, whereas the conditional residual variability increased substantially. At paired means of 15%, 20%, and 25%, the estimated mean differences were −4.17, −4.21, and −4.25 percentage points (pp), respectively, while the corresponding conditional SDs were 0.90, 1.56, and 2.39 pp. The resulting level-specific 95% LoA were −5.93 to −2.41 pp at a paired mean of 15%, −7.26 to −1.16 pp at 20%, and −8.93 to 0.44 pp at 25%. Participant-level bootstrap analysis with 10,000 replicates yielded 95% CIs of −6.47 to −5.40 and −3.32 to −1.61 pp for the lower and upper LoA, respectively, at 15%; −8.03 to −6.45 and −2.45 to −0.16 pp at 20%; and −11.60 to −6.92 and −2.40 to 2.72 pp at 25% (Supplementary Table S3).

**Figure 2:**
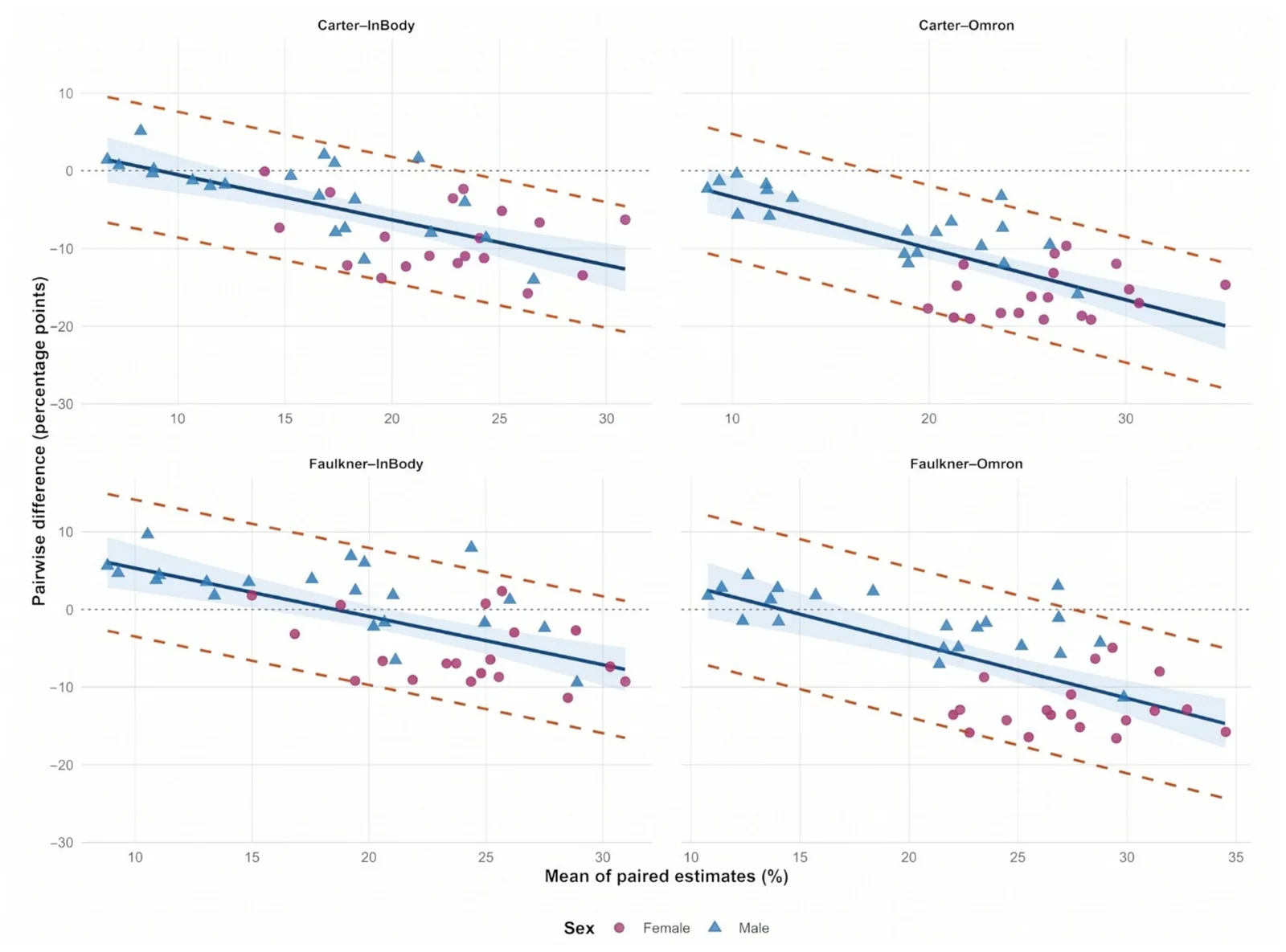
Level-dependent differences between anthropometric and BIA body-fat estimates. Difference-versus-paired-mean plots for the Carter–InBody, Carter–Omron, Faulkner–InBody, and Faulkner–Omron comparisons. The solid blue line represents the fitted conditional mean difference, the orange dashed lines represent the regression-based 95% limits of agreement, and the blue shaded band represents the 95% confidence interval for the conditional mean difference. The horizontal dotted line indicates zero pairwise difference. Circles represent women and triangles represent men. Conditional estimates are descriptive and do not establish accuracy or criterion validity.

The fitted slope for the conditional mean difference was small (−0.0076 pp per percentage-point increase in paired mean; bootstrap 95% CI, −0.0838 to 0.0510), whereas the selected variance model indicated increasing residual dispersion across the paired-mean range. Thus, the principal non-constant feature of the Carter–Faulkner comparison was the variance rather than the mean structure. This pattern is illustrated in Figure 2, in which the fitted mean difference remains nearly horizontal while the level-specific LoA widen across the paired-mean range. The classical LoA of −7.04 to −1.27 pp are retained as a global descriptive summary but do not characterize this changing dispersion across the measurement range as adequately as the heteroscedastic model.

### 3.4. Difference-on-Paired-Mean Patterns and Mathematical-Coupling Audit

Difference-on-paired-mean regressions showed negative slopes for each of the four anthropometry–BIA comparisons: −0.662 for Carter–Omron, −0.582 for Carter–InBody, −0.721 for Faulkner–Omron, and −0.622 for Faulkner–InBody. In the observed data, therefore, the differences became more negative as the corresponding paired mean increased. However, these associations were reassessed considering the mathematical coupling between the paired difference and paired mean.

The anthropometry-derived estimates showed substantially lower between-participant variability than the BIA estimates. SDs were 4.85 pp for Carter-derived estimates and 4.74 pp for Faulkner-derived estimates, compared with 8.99 pp for Omron and 8.27 pp for InBody. Consequently, the anthropometry-to-BIA variance ratios ranged from approximately 0.28 to 0.34. Across all six pairwise comparisons, the directly estimated difference-on-paired-mean slopes agreed, to numerical precision, with the slopes algebraically implied by the marginal variances and covariance structure of the same paired measurements (Table 3).

This agreement indicates that the observed slopes cannot be treated as statistically independent evidence that between-method disagreement changes with underlying true body-fat level. Rather, they describe the geometry of the observed paired measurements. Without a criterion measure, replicate measurements, or independently estimated method-specific error variances, the present data cannot distinguish a genuine adiposity-level relationship from the statistical structure of the paired measurements.

**Figure 3:**
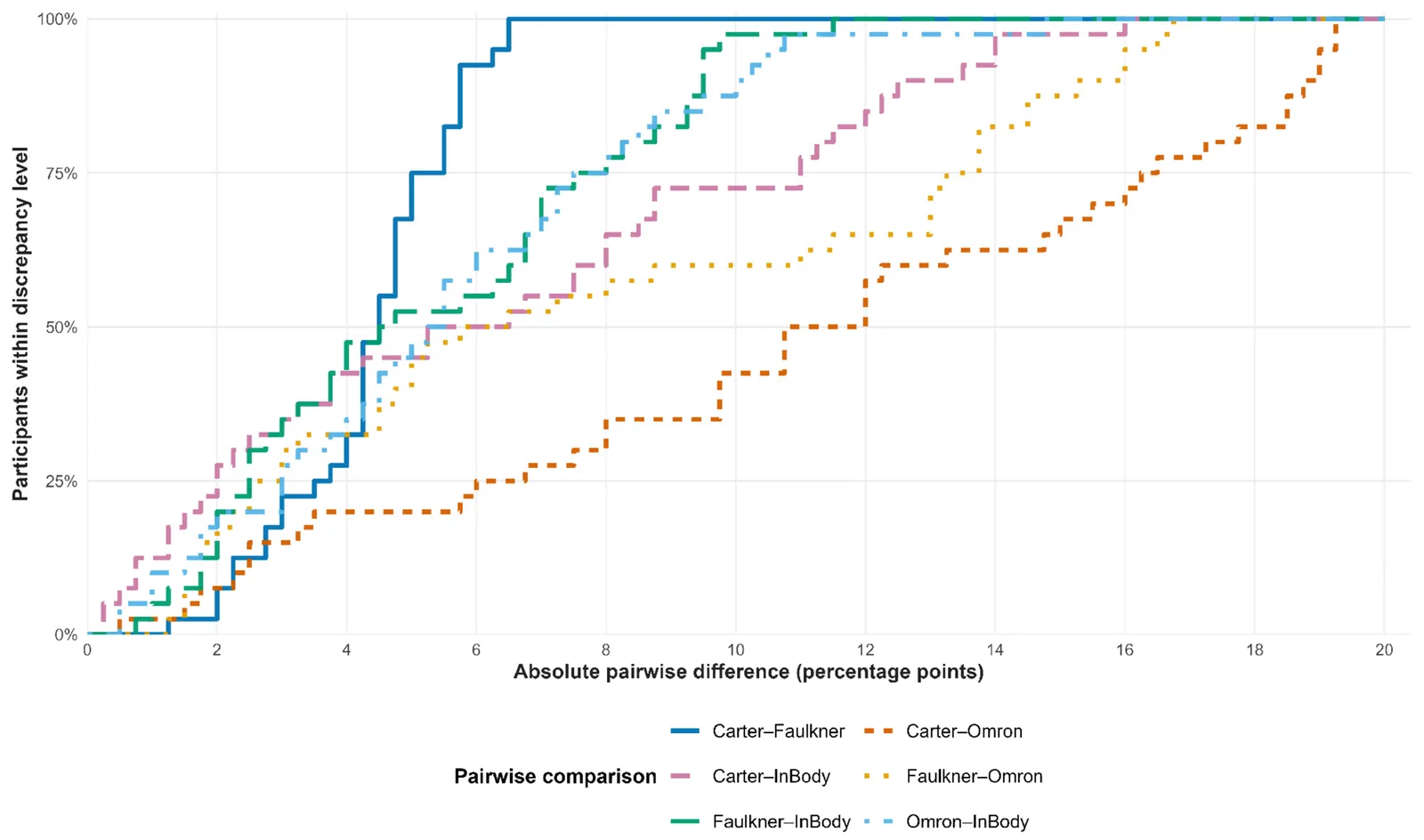
Descriptive relationship between paired differences and paired means for anthropometry–BIA comparisons. Difference-versus-paired-mean plots for Carter–Omron, Carter–InBody, Faulkner–Omron, and Faulkner–InBody. Differences were calculated as the first-listed method minus the second-listed method. Solid lines represent fitted conditional mean differences. Because the paired difference and paired mean are mathematically coupled and the compared methods have unequal marginal variances, the fitted slopes are presented descriptively and should not be interpreted as independent evidence that disagreement changes with underlying true body-fat level.

Carter–Faulkner, for which the marginal SDs were similar (4.85 and 4.74 pp), showed a difference-on-paired-mean slope close to zero (0.024), whereas Omron–InBody, which also had more similar marginal SDs (8.99 and 8.27 pp), showed a comparatively small positive slope (0.087). For completeness, descriptive conditional differences predicted at paired means of 15%, 20%, and 25% are reported in Supplementary Table S4. These predictions derive from the same mathematically coupled regressions and are therefore not interpreted as estimates of disagreement at known levels of true adiposity. The observed difference-on-paired-mean relationships for the four anthropometry–BIA comparisons are illustrated in Figure 3.

### 3.5. Secondary and Sensitivity Analyses

Secondary agreement summaries reinforced the distinction between group-level numerical similarity and participant-level disagreement. Absolute-difference distributions and complementary ICC estimates are reported in Supplementary Table S5. ICCs were interpreted as measures of consistency rather than as substitutes for Bland–Altman agreement.

Exploratory sex-stratified analyses and models adjusted for sex and age are presented in Supplementary Table S6. Sex-stratified Bland–Altman summaries are illustrated in Supplementary Figure S2, while crude and sex- and age-adjusted difference-on-paired-mean slopes are compared in Supplementary Figure S4. Adjustment attenuated the crude difference-on-paired-mean slopes by approximately 19% to 55% across the four anthropometry–BIA comparisons. Given the small sex-specific sample sizes, the relationship between sex and the distribution of body-fat estimates, and the mathematical coupling inherent in the difference-on-paired-mean regressions, these analyses were considered descriptive and were not used to infer sex-specific method effects or an independent body-fat-level effect.

Deming regression sensitivity analyses across alternative assumed error-variance ratios are reported in Supplementary Table S7. Estimated method-to-method relationships varied according to the assumed relative error variance. Because the study contained neither replicate measurements nor external estimates of method-specific measurement-error variance, no single error-variance ratio could be empirically identified. The Deming analyses were therefore interpreted as assumption-based sensitivity analyses rather than as a definitive correction for measurement error.

Influence diagnostics identified several observations exceeding the conventional Cook’s-distance threshold; however, leave-one-participant-out analyses produced only small changes in the estimated mean differences and LoA, and no single exclusion materially altered the overall interpretation of the principal agreement results (Supplementary Figure S5 and Supplementary Table S8). Additional analyses of LoA precision and sample-size sensitivity are reported in Supplementary Table S9 and illustrate the uncertainty associated with the present LoA estimates.

## 4. Discussion

The present study characterized pairwise numerical agreement among four field-based methods used to estimate body-fat percentage in physically active adults, without designating any method as a criterion reference. Three findings are central. First, systematic numerical differences were observed across several method pairs, and modest differences in group means did not necessarily correspond to close agreement at the individual level. Second, the Carter–Faulkner comparison showed an approximately stable mean difference but increasing residual dispersion across the measurement range. Third, although all four anthropometry–BIA comparisons showed negative difference-on-paired-mean slopes, these slopes were algebraically consistent with the unequal marginal variances of the compared methods and therefore cannot be interpreted as independent evidence that disagreement changes with underlying true adiposity.

The distinction between average numerical difference and individual-level agreement is particularly important. Carter-derived estimates averaged 4.16 percentage points (pp) lower than Faulkner-derived estimates, whereas Omron estimates averaged 5.34 pp higher than InBody estimates. However, Faulkner–InBody showed a relatively small mean difference of −1.48 pp despite global 95% limits of agreement (LoA) extending from −12.91 to 9.94 pp. Thus, similarity in group averages did not necessarily imply close agreement for individual participants.

This distinction is consistent with established method-comparison principles. Correlation, consistency, or similarity of mean values cannot substitute for assessment of agreement, and LoA quantify the range within which most differences between paired measurements are expected to occur [11,12,13]. Whether that disagreement is sufficiently small for practical use requires comparison with an acceptable difference defined according to the intended application [11,13]. Because no application-specific acceptability threshold was specified a priori in the present study, the observed LoA quantify disagreement but do not establish interchangeability or non-interchangeability.

The Carter–Faulkner comparison should be interpreted specifically as a comparison between the two archived anthropometry-derived estimates examined in this study rather than as evidence about anthropometric methods in general. Previous research has shown that skinfold-derived body-composition estimates can differ according to equation specification, anatomical sites, sex, sport, adiposity, and characteristics of the derivation population [6,7,18,19,20]. Contemporary evidence consequently emphasizes that predictive equations should be selected with consideration of the population and body-composition model for which they were developed [6,7].

This issue is relevant to the historical context of the Carter- and Faulkner-derived estimates. Published descriptions of these approaches indicate that they emerged from specific athletic contexts and differ in the skinfold information incorporated into the prediction procedure [14,17]. These characteristics provide a plausible context for why two anthropometry-derived estimates should not automatically be expected to produce numerically equivalent body-fat percentages. They do not, however, establish the cause of the difference observed in the present sample.

Importantly, the complete computational workflow used to generate the archived Carter- and Faulkner-labeled variables was not retained. The exact historical equation implementation and intermediate calculations therefore cannot be independently reconstructed from the analytical dataset. Retrospectively applying equations reported in the literature would not demonstrate that those equations were identical to those originally implemented. Accordingly, the present results should be interpreted as agreement between archived Carter-derived and Faulkner-derived outputs rather than as a validation or recalibration of the original equations. This limitation restricts mechanistic attribution of the observed −4.16-pp mean difference and directly addresses the reproducibility uncertainty inherent in these archived variables.

Instrumentation represents an additional source of uncertainty for anthropometric estimates. Skinfold thicknesses were measured using a Cescorf Innovare caliper, whereas historical equations may have been developed using other instruments. Skinfold calipers can differ in mechanical characteristics, including jaw geometry and applied pressure, and instrument-related variation may affect recorded skinfold thickness [2,16]. Nevertheless, the present study did not directly compare calipers, and measurements were obtained using standardized ISAK procedures with technical errors of measurement within the study’s predefined quality thresholds. The contribution of the specific caliper to the observed estimates therefore cannot be quantified and should be considered a potential source of measurement uncertainty rather than an established explanation for the Carter–Faulkner difference.

The comparison between Omron and InBody similarly demonstrates that two devices belonging to the broad category of BIA should not be assumed to produce equivalent estimates. Omron estimates averaged 5.34 pp higher than InBody estimates, with global LoA ranging from −1.46 to 12.13 pp. BIA devices may differ in current frequency, electrode configuration, body pathway, measurement implementation, and the prediction procedures used to transform electrical measurements into body-composition estimates [3,5,8,9,21,22].

Recent evidence reinforces the importance of these device-specific characteristics. Bennett et al. demonstrated variation in raw bioimpedance measurements across devices in athletes, with subsequent effects on predicted body-composition estimates [8]. Similarly, comparisons across different bioimpedance technologies indicate that agreement depends on the particular technologies and prediction procedures being compared rather than simply on their common classification as BIA [21]. The present Omron–InBody findings should therefore be interpreted as specific to these two devices and should not be generalized to single-frequency versus multifrequency BIA systems as categories.

The proprietary nature of commercial BIA algorithms introduces an additional limitation. Prediction models may incorporate demographic and anthropometric variables in addition to measured electrical properties, while the complete equations and development datasets are not necessarily available for independent evaluation [4,5,9,22]. Consequently, uncertainty regarding derivation populations and model transportability is not exclusive to anthropometric equations. The numerical disagreement observed between Omron and InBody may reflect differences in electrical measurement, prediction algorithms, or both, and the present design cannot separate these components.

Pre-assessment conditions may also have contributed to participant-level variation. BIA is sensitive to assumptions concerning body water and fluid distribution, and recent exercise, food and fluid intake, hydration status, posture, and other measurement conditions can affect impedance or derived estimates [4,5,10,23,24]. Cataldi et al. specifically demonstrated the relevance of hydration when evaluating multiple body-composition methods in athletes [3], while methodological standards emphasize careful standardization when BIA is used in research or clinical assessment [4].

Participants in the present study received standardized instructions regarding exercise, fasting, alcohol and tobacco consumption, fluid intake, and bladder emptying. However, adherence and hydration status were not objectively verified. Consequently, part of the observed participant-level variability may reflect uncontrolled biological or pre-assessment variability rather than differences attributable exclusively to the devices. The same-session design reduces between-session variation but does not eliminate these sources of uncertainty.

A central methodological issue concerns the negative difference-on-paired-mean slopes observed in the four anthropometry–BIA comparisons. For a paired difference *D* = *A* − *B* and paired mean (*M* = (*A* + *B*)/2), the relationship (*Cov*(*D*,*M*) = [*Var*(*A*) − *Var*(*B*)]/2) shows that the difference and paired mean are mathematically related whenever the marginal variances of the two methods differ. Mathematical relationships involving differences and quantities constructed from the original measurements have long been recognized as a potential source of misleading associations in method-comparison analyses [14].

In the present data, the BIA estimates showed substantially greater between-participant variability than the anthropometry-derived estimates. Moreover, the observed difference-on-paired-mean slopes agreed to numerical precision with the slopes algebraically implied by the corresponding marginal variances and covariance structure. The negative slopes therefore describe the geometry of the observed paired measurements but cannot provide independent evidence that anthropometric estimates become progressively lower than BIA estimates as true adiposity increases.

This reinterpretation is important because the initial descriptive pattern could otherwise be mistaken for proportional or adiposity-dependent disagreement. Bland–Altman extensions allow systematic changes in mean differences or variability across the measurement range to be modelled when appropriate [11,25,26], but interpretation of such patterns must distinguish empirical heteroscedasticity from associations generated by the mathematical construction of the plotted variables [14]. In the absence of an external criterion or independently estimated measurement-error components, the present data cannot determine whether a genuine relationship with underlying adiposity exists beyond the observed variance structure.

Adjustment for sex and age does not resolve this identification problem. The adjusted analyses attenuated the crude slopes, but statistical adjustment cannot remove the mathematical coupling between the difference and paired mean. Furthermore, body-fat distributions differed by sex within this relatively small sample. The data therefore cannot cleanly separate a relationship with adiposity magnitude from possible sex-related differences in method behavior. This is consistent with evidence that body-composition estimates and agreement across methods can differ between women and men [26].

The Deming regression sensitivity analyses likewise cannot provide a definitive correction. Deming regression requires information or assumptions about the relative measurement-error variance of the methods being compared. Because replicate measurements and independent estimates of method-specific error variance were unavailable, no single error-variance ratio could be empirically identified. Variation in the estimated relationships across the assumed ratios should therefore be interpreted as sensitivity to modelling assumptions rather than evidence of proportional disagreement.

The Carter–Faulkner comparison presented a different phenomenon. The fitted conditional mean difference remained approximately −4.2 pp at paired means of 15%, 20%, and 25%, whereas the conditional SD increased from 0.90 to 2.39 pp. Consequently, the level-specific LoA widened as the paired mean increased. The principal non-constant feature of this comparison was therefore the variance rather than the mean structure.

This distinction is methodologically important. Bland and Altman recognized that constant-width LoA may provide an inadequate representation when the variability of paired differences changes with measurement magnitude [13]. More recent methodological work similarly emphasizes that conventional Bland–Altman assumptions should be examined rather than applied mechanically when bias or precision is non-constant [11,25]. The classical Carter–Faulkner LoA therefore remain useful as an overall descriptive summary, whereas the heteroscedastic model more accurately represents the changing dispersion observed in this sample.

The widening bootstrap confidence intervals for the level-specific Carter–Faulkner LoA also highlight the uncertainty associated with these conditional estimates, particularly toward the upper evaluated range. This finding should not be interpreted as evidence that the equations themselves become systematically more biased with increasing adiposity. Rather, the observed data indicate increasing dispersion in their pairwise differences. Larger samples covering a broader body-composition range would be required to estimate this variance structure more precisely [27].

From an applied perspective, the results indicate that estimates generated by different field methods should not automatically be treated as values on a common numerical scale. This is particularly relevant in sport and exercise settings, where body-composition measurements may be repeated to support nutritional assessment, training decisions, or longitudinal monitoring [1,2]. Existing evidence demonstrates that both equation selection and BIA technology can influence the resulting body-composition estimates [6,8,20,21,24].

The present study does not establish that any method pair is interchangeable or non-interchangeable, nor does it identify one method as more accurate than another. Accuracy requires an appropriate criterion method, whereas interchangeability additionally requires an application-specific definition of acceptable disagreement [11,12,13]. Nevertheless, the systematic and participant-specific differences observed here indicate that longitudinal comparisons involving a change of equation or device should be interpreted cautiously. Consistent use of the same assessment approach and standardized measurement conditions is therefore important when serial measurements are intended to represent biological change [1,2,24].

Several features strengthen the study. All four estimates were obtained from the same participants during the same assessment session, reducing between-session biological variation and allowing direct examination of all six pairwise comparisons. The analysis deliberately avoided designating any field method as a criterion reference and focused on participant-level differences and LoA rather than relying on correlation alone [11,12,13]. Bootstrap uncertainty estimates, explicit modeling of non-constant dispersion, influence analyses, measurement-error sensitivity analyses, and the mathematical-coupling audit further allowed important assumptions underlying the agreement analyses to be examined.

Several limitations define the boundaries of inference. The sample comprised 40 physically active adults, resulting in appreciable uncertainty around several LoA and limiting sex-specific analyses. No criterion or multicomponent body-composition method was included; consequently, the study evaluates numerical agreement rather than accuracy, and the direction of error relative to true adiposity cannot be established [3,7,28]. Replicate measurements were also unavailable, preventing separation of within-method measurement error from between-method disagreement and empirical estimation of method-specific error variances.

The archived nature of the Carter- and Faulkner-derived variables represents an additional reproducibility limitation. The complete calculation workflow and exact equation implementation were not retained, and the possible influence of the Cescorf Innovare caliper relative to instruments used in historical equation-development studies cannot be quantified. For BIA, hydration status and adherence to pre-assessment instructions were not objectively verified. These limitations prevent the observed differences from being attributed uniquely to the underlying prediction equations or device technologies.

Menstrual-cycle phase was not systematically recorded. Cycle-related changes in body weight and body composition have been reported [29], although recent repeated-measures evidence suggests that reliability of body-composition assessments may remain high across menstrual-cycle phases under some conditions [30]. Its contribution to the present findings therefore cannot be determined, and the observed sex-related patterns should not be retrospectively attributed to menstrual-cycle variation.

The environmental context also warrants consideration. Measurements were obtained in Quito at approximately 2850 m above sea level, but habitual residence and individual acclimatization were not systematically recorded. Altitude exposure can affect body weight and body-composition-related variables, with effects depending on altitude, duration of exposure, physical activity, energy balance, and acclimatization [31]. Because all four methods were applied to each participant under the same environmental conditions, altitude cannot be identified as the cause of the observed pairwise differences. Instead, unmeasured acclimatization primarily limits generalizability to populations assessed under different environmental conditions.

Future studies should combine larger samples spanning deliberately broad ranges of body composition with replicate measurements for each method. Replication would allow method-specific measurement error to be estimated and would strengthen measurement-error modeling. Inclusion of an appropriate multicomponent criterion method would additionally permit accuracy to be evaluated rather than agreement alone [4,7,28,32].

Complete retention of raw anthropometric measurements, raw impedance variables, prediction equations, device and software versions, and intermediate calculations should also be prioritized. Such designs would improve reproducibility and help distinguish disagreement attributable to measurement instrumentation from disagreement introduced by prediction models. Future studies should additionally define application-specific acceptable differences before analysis so that observed LoA can be interpreted against an explicit practical criterion [11,12,13].

## 5. Conclusions

The four field-based body-fat estimation methods examined in this study showed systematic pairwise numerical differences and, for several comparisons, substantial individual-level disagreement. A small mean difference did not necessarily indicate close agreement between methods, emphasizing the importance of considering the distribution and uncertainty of participant-level differences rather than group averages alone.

The Carter–Faulkner comparison was characterized by an approximately stable mean difference but increasing dispersion across the measurement range, supporting the use of level-specific LoA to complement the global agreement summary. In contrast, the negative difference-on-paired-mean slopes observed in the anthropometry–BIA comparisons were consistent with mathematical coupling and unequal method variances and therefore cannot be interpreted as independent evidence that disagreement increases with true adiposity.

These findings are specific to the methods and population examined and should not be generalized to anthropometry or BIA as methodological categories. Because the study included no criterion method, replicate measurements, or prespecified application-specific acceptability threshold, it does not establish accuracy, superiority, or interchangeability among the methods. Future method-comparison studies should incorporate replicate measurements, appropriate criterion methods when accuracy is of interest, complete retention of measurement and computational workflows, and prospectively defined acceptability criteria.

## Figures and Tables

**Table 1:** Characteristics of the study participants.

Characteristic	Overall (n = 40)
Age, years	34.8 ± 8.8
**Sex, n (%)**	
Female	19 (47.5)
Male	21 (52.5)
**Sport or physical-activity group, n (%)**	
Athletics	14 (35.0)
Mountaineering	10 (25.0)
CrossFit	6 (15.0)
Triathlon	4 (10.0)
Walking	3 (7.5)
Weightlifting	2 (5.0)
Soccer	1 (2.5)

Note: Data are presented as mean ± standard deviation or n (%), as appropriate. Percentages were calculated using the total sample (n = 40) as the denominator.

**Table 2:** Pairwise differences and classical Bland–Altman agreement parameters for body-fat estimates.

Pairwise Comparison	Mean Difference, pp (95% CI)	SD of Differences, pp	Global 95% LoA, pp	Variance Structure
Carter–Faulkner	−4.16 (−4.59 to −3.68)	1.47	−7.04 to −1.27	Non-constant *
Carter–Omron	−10.98 (−12.82 to −9.14)	5.97	−22.67 to 0.72	Constant
Carter–InBody	−5.64 (−7.30 to −3.97)	5.45	−16.32 to 5.04	Constant
Faulkner–Omron	−6.82 (−8.86 to −4.77)	6.69	−19.93 to 6.29	Constant
Faulkner–InBody	−1.48 (−3.26 to 0.32)	5.83	−12.91 to 9.94	Constant
Omron–InBody	5.34 (4.28 to 6.42)	3.47	−1.46 to 12.13	Constant

Note: Differences were calculated as the first-listed method minus the second-listed method. * For Carter–Faulkner, a varPower model was favored over the constant-variance model (ΔAIC = −12.86); level-specific LoA are reported in Supplementary Table S3. No method was treated as a criterion reference, and no application-specific acceptability threshold was prespecified. Abbreviations: CI, confidence interval; LoA, limits of agreement; pp, percentage points; SD, standard deviation.

**Table 3:** Difference-on-paired-mean slopes and mathematical-coupling diagnostics for pairwise body-fat comparisons.

Pairwise Comparison	SD Method A, pp	SD Method B, pp	Variance Ratio (A/B)	Observed D-on-M Slope	Algebraically Implied Slope *
Carter–Faulkner	4.85	4.74	1.05	0.024	0.024
Carter–Omron	4.85	8.99	0.29	−0.662	−0.662
Carter–InBody	4.85	8.27	0.34	−0.582	−0.582
Faulkner–Omron	4.74	8.99	0.28	−0.721	−0.721
Faulkner–InBody	4.74	8.27	0.33	−0.622	−0.622
Omron–InBody	8.99	8.27	1.18	0.087	0.087

Note: A and B correspond to the first- and second-listed methods, respectively. For each participant, the paired difference was defined as *D* = *A* − *B* and the paired mean as *M* = (*A* + *B*)/2. SD, standard deviation; pp, percentage points. The variance ratio was calculated as *Var*(*A*)/*Var*(*B*). * The algebraically implied difference-on-paired-mean slope was obtained from the identity *Cov*(*M*) = [*Var*(*A*) − *Var*(*B*)]/2, together with *β_D_*_|*M*_ = *Cov*(*M*)/*Var*(*M*). Observed and algebraically implied slopes agreed to numerical precision. Accordingly, the D-on-M slopes are not statistically independent of the marginal variances of the two methods and should not be interpreted by themselves as evidence that between-method disagreement changes with the underlying true body-fat level. Carter-derived and Faulkner-derived values refer to the archived anthropometry-derived estimates labelled as Carter and Faulkner in the original dataset. No method was treated as a criterion reference.

## Data Availability

The data supporting the findings of this study are available from the corresponding author upon reasonable request. The data are not publicly available because they contain individual-level information from human participants and their public release was not included in the original informed consent procedure. Any data sharing will be subject to approval by the corresponding author and compliance with applicable ethical and confidentiality requirements. The analytical code used to reproduce the statistical analyses is available from the corresponding author upon reasonable request.

## Supplementary Materials

The following supporting information can be downloaded at: https://www.mdpi.com/article/10.3390/jfmk11040401/s1, Figure S1: Mean pairwise differences and classical limits of agreement across body-fat assessment methods; Figure S2: Exploratory sex-stratified Bland–Altman summaries; Figure S3: Conventional Bland–Altman plots for all six pairwise body-fat comparisons; Figure S4: Sex- and age-adjusted sensitivity analyses of difference-on-paired-mean associations; Figure S5: Influence diagnostics for difference-on-paired-mean models; Table S1: Descriptive distributions of body-fat estimates across the four assessment methods; Table S2: Comparison of constant and non-constant residual variance models; Table S3: Level-specific limits of agreement for the Carter–Faulkner comparison under the selected heteroscedastic variance model; Table S4: Descriptive conditional differences across selected paired-mean values; Table S5: Absolute pairwise differences and intraclass correlation coefficients; Table S6: Exploratory sex-stratified and sex- and age-adjusted sensitivity analyses; Table S7: Deming regression sensitivity analyses across assumed error-variance ratios; Table S8: Influence diagnostics and leave-one-participant-out robustness analyses; Table S9: Precision of limits of agreement in the current sample and illustrative sample-size requirements for future studies.

## Abbreviations

The following abbreviations are used in this manuscript:

**Abbreviation**	**Definition**
**AIC**	Akaike information criterion
**BIA**	Bioelectrical impedance analysis
**CI**	Confidence interval
**DXA**	Dual-energy X-ray absorptiometry
**ECDF**	Empirical cumulative distribution function
**ICC**	Intraclass correlation coefficient
**IQR**	Interquartile range
**ISAK**	International Society for the Advancement of Kinanthropometry
**LoA**	Limits of agreement
**pp**	Percentage points
**SD**	Standard deviation
**STROBE**	Strengthening the Reporting of Observational Studies in Epidemiology
**TEM**	Technical error of measurement
