# Supplementary material for: Pairwise Agreement Among Field-Based Body-Fat Estimation Methods in Physically Active Adults: A Cross-Sectional Method-Comparison Study
Source: J Funct Morphol Kinesiol. 2026 Oct 2;11(4):401. doi: 10.3390/jfmk11040401 (PMC13692335; doi:10.3390/jfmk11040401)
Supplement: Supplementary file 1 [file jfmk-11-00401-s001.zip › jfmk-4569822-supplementary.pdf]

Supplementary Materials

Pairwise Agreement Among Field-Based Body-Fat Estimation Methods in Physically Active Adults: A Cross-Sectional Method-Comparison Study

Supplementary Figures

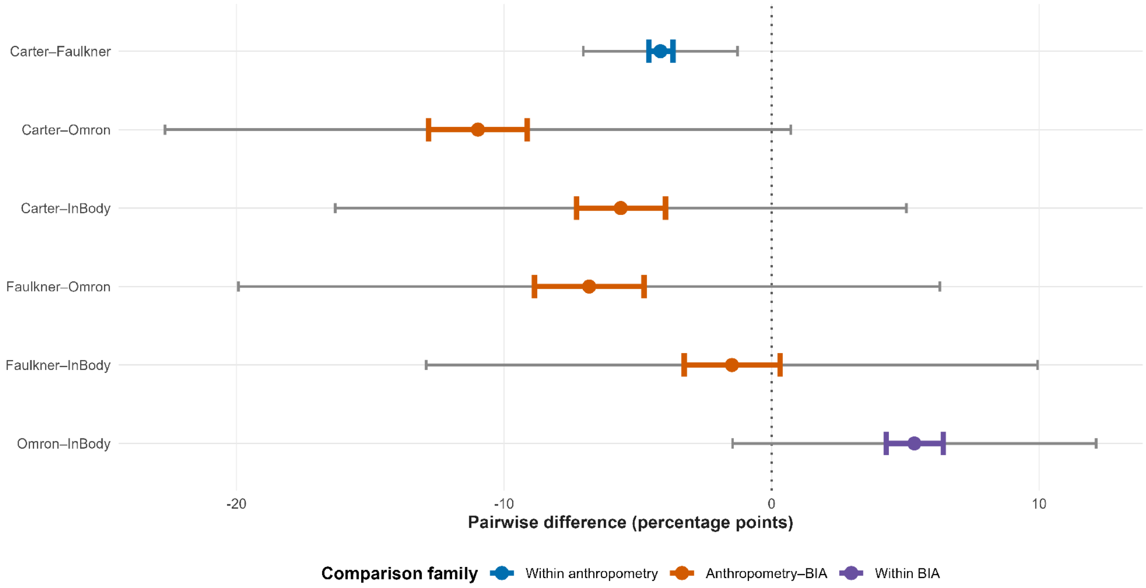

Supplementary Figure S1. Mean pairwise differences and classical limits of agreement across body-fat assessment methods. Points represent mean paired differences, thick intervals represent participant-bootstrap 95% confidence intervals for the mean difference, and thin intervals represent classical 95% limits of agreement. Differences are defined as the first-listed method minus the second-listed method. The vertical reference line denotes zero difference. Pair labels identify the specific comparisons examined; no method was treated as a criterion measure.

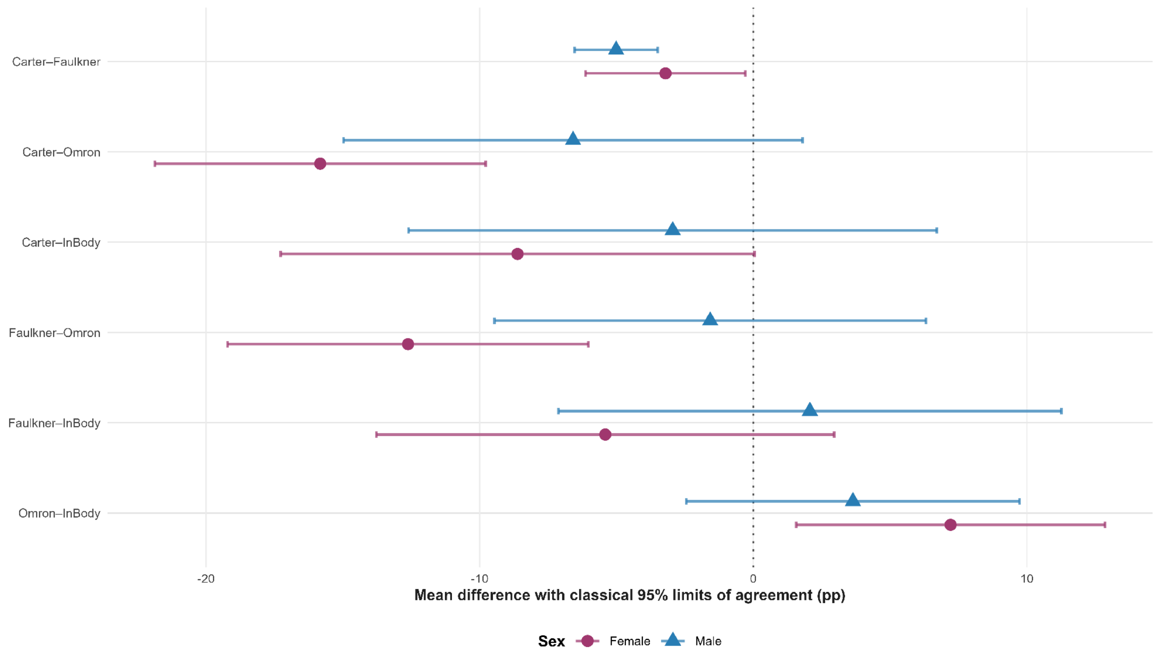

Supplementary Figure S2. Exploratory sex-stratified Bland–Altman summaries. Mean paired differences and classical 95% limits of agreement are shown separately for women and men for all six pairwise comparisons. These analyses are descriptive and hypothesis-generating; no confirmatory method-by-sex interaction testing was performed.

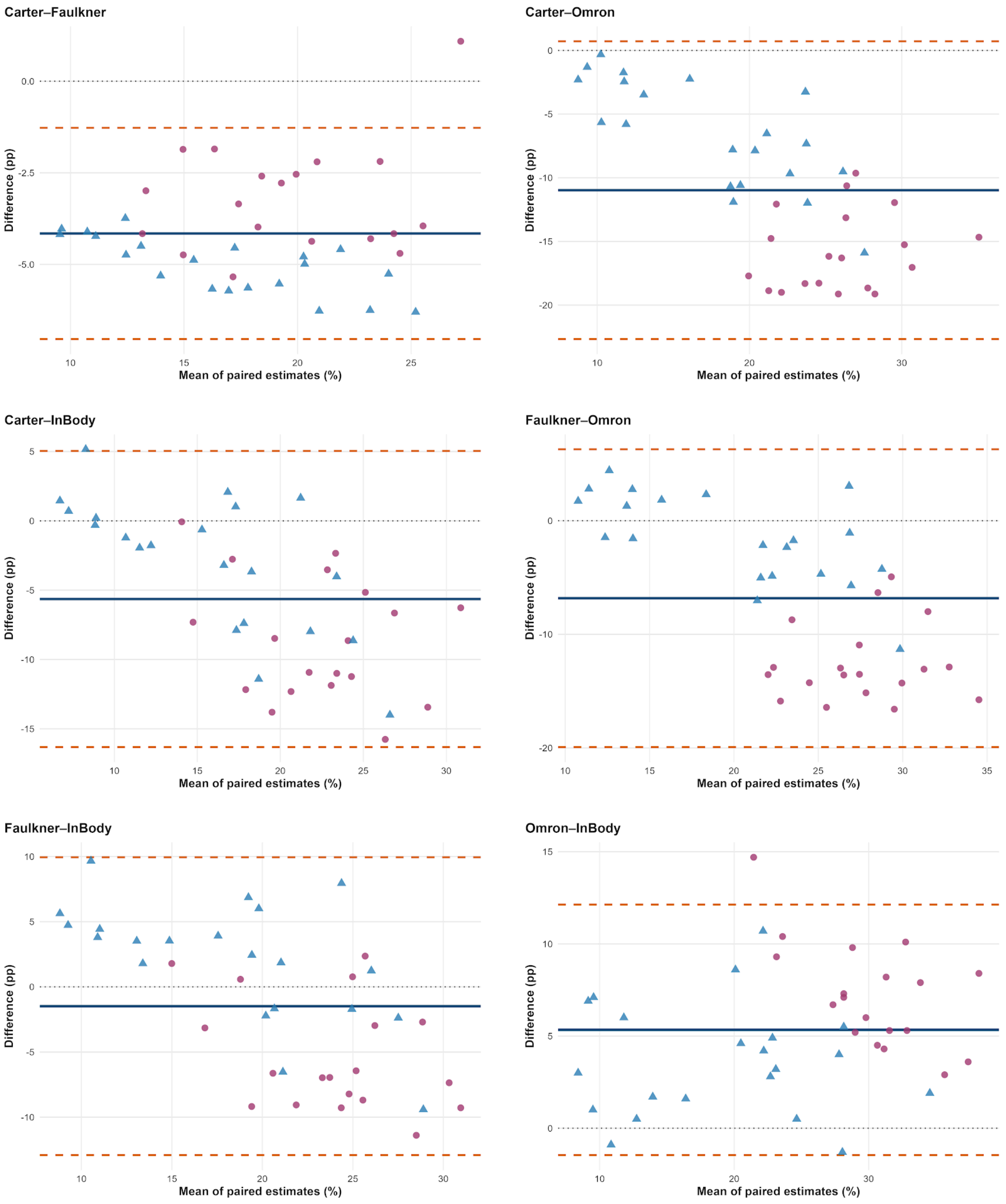

Supplementary Figure S3. Conventional Bland–Altman plots for all six pairwise body-fat comparisons. Participant-level paired differences are plotted against the corresponding paired means. The solid line represents the overall mean difference, dashed lines represent the classical 95% limits of agreement, and the horizontal reference line denotes zero difference. These plots are descriptive displays of the observed paired measurements. Associations between paired differences and paired means should not be interpreted as independent evidence of magnitude-dependent disagreement because both quantities are constructed from the same measurements and may be mathematically coupled when method variances differ. Point colour and shape distinguish women and men for descriptive visualization only.

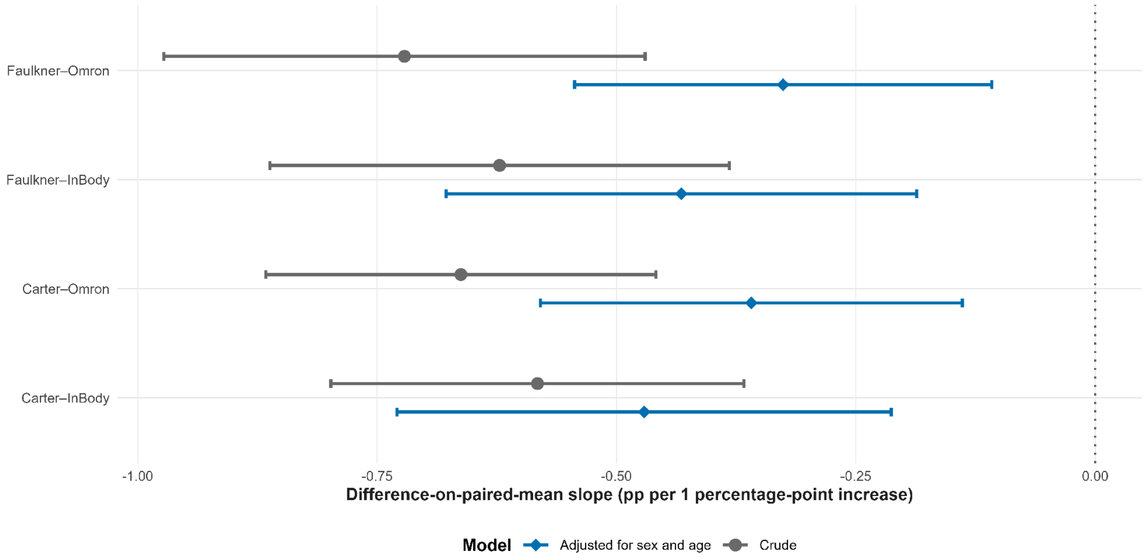

Supplementary Figure S4. Sex- and age-adjusted sensitivity analyses of difference-on-paired-mean associations. Crude difference-on-paired-mean slopes are compared with slopes from models adjusted for sex and age for the four anthropometry–BIA comparisons. Horizontal intervals represent 95% confidence intervals. These analyses were descriptive sensitivity analyses. Adjustment does not eliminate mathematical coupling between paired differences and paired means; consequently, the estimates were not interpreted as evidence of an independent adiposity-level effect or as confirmatory evidence of effect modification.

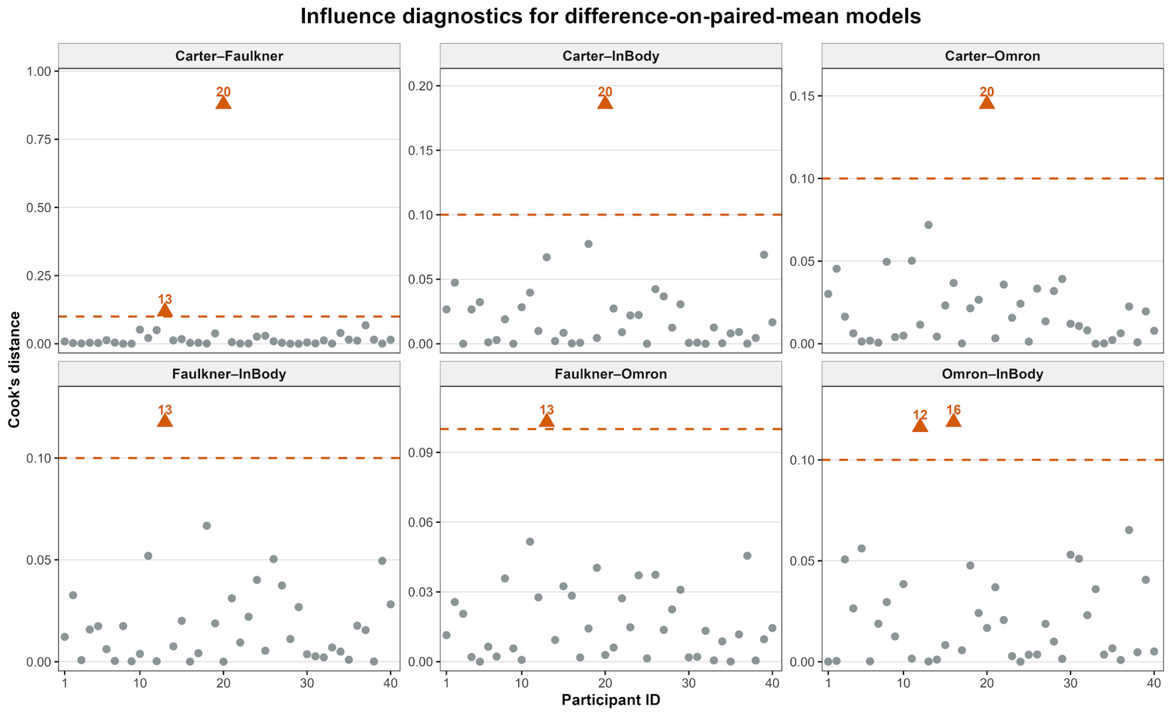

Supplementary Figure S5. Influence diagnostics for difference-on-paired-mean models. Cook's distance is shown for each participant in each pairwise comparison. The dashed horizontal line represents the conventional 4/n screening threshold (0.10 for n = 40). Triangles identify observations exceeding this diagnostic threshold. No observation was excluded solely on this basis; robustness was assessed using leave-one-participant-out analyses reported in Supplementary Table S8.

Supplementary Tables

Supplementary Table S1. Complete descriptive distributions of body-fat estimates by assessment method.

Method	n	Mean, %	SD, pp	Median, %	IQR, %	Minimum, %	Maximum, %
Carter-derived anthropometry	40	16.04	4.85	16.00	12.41–19.64	7.45	27.73
Faulkner-derived anthropometry	40	20.20	4.74	20.04	16.45–24.11	11.63	28.35
Omron HBF-514C	40	27.02	8.99	28.80	23.77–33.40	9.90	42.40
InBody 120	40	21.68	8.27	23.95	15.75–27.88	5.70	35.60

Note: All four method-specific body-fat outputs were complete for the 40 participants. IQR, interquartile range; pp, percentage points; SD, standard deviation.

Supplementary Table S2. Comparison of constant and non-constant residual variance models for the six pairwise comparisons.

Comparison	Constant-SD AIC	varPower AIC	ΔAIC	varExp AIC	ΔAIC	Selected variance structure
Carter–Faulkner	149.15	136.29	-12.86	138.11	-11.04	varPower
Carter–Omron	230.96	228.79	-2.16	230.25	-0.71	Constant variance
Carter–InBody	230.91	227.70	-3.21	229.13	-1.78	Constant variance
Faulkner–Omron	245.07	242.86	-2.21	244.41	-0.66	Constant variance
Faulkner–InBody	237.71	235.09	-2.62	236.57	-1.15	Constant variance
Omron–InBody	216.15	217.94	1.79	217.49	1.34	Constant variance

Note: Models were fitted using maximum likelihood. ΔAIC was calculated relative to the constant-variance model; negative values favor the corresponding non-constant variance specification. A reduction of ≥4 AIC units was used as the model-selection criterion. Carter–Faulkner was the only comparison meeting this criterion, with varPower providing the best-fitting variance structure (ΔAIC = −12.86).

Supplementary Table S3. Level-specific limits of agreement for the Carter–Faulkner comparison under the selected heteroscedastic variance model.

Paired mean	Predicted difference, pp	Bootstrap 95% CI	Conditional SD, pp	Bootstrap 95% CI	Lower LoA, pp	Bootstrap 95% CI	Upper LoA, pp	Bootstrap 95% CI
15%	-4.17	-4.51 to -3.84	0.90	0.57 to 1.21	-5.93	-6.47 to -5.40	-2.41	-3.32 to -1.61
20%	-4.21	-4.85 to -3.66	1.56	1.10 to 1.89	-7.26	-8.03 to -6.45	-1.16	-2.45 to -0.16
25%	-4.25	-5.22 to -3.42	2.39	1.22 to 3.55	-8.93	-11.58 to -6.92	0.44	-2.40 to 2.72

Note: Values are expressed in percentage points (pp). Conditional mean differences and 95% limits of agreement (LoA) were estimated from the selected generalized least-squares model with a linear mean structure and a varPower residual variance function of the paired mean. Confidence intervals were obtained using a participant-level percentile bootstrap with 10,000 replicates; all 10,000 bootstrap samples produced successful model fits. Differences were calculated as Carter-derived minus Faulkner-derived body-fat percentage. The evaluated paired means (15%, 20%, and 25%) are representative values within the observed measurement range and do not represent known levels of true adiposity.

Supplementary Table S4. Descriptive conditional difference-on-paired-mean estimates at selected paired means for anthropometry–BIA comparisons.

Comparison	Paired mean 15%, pp	Paired mean 20%, pp	Paired mean 25%, pp
Carter–Omron	-6.65	-9.96	-13.28
Carter–InBody	-3.39	-6.30	-9.22
Faulkner–Omron	-0.61	-4.22	-7.83
Faulkner–InBody	2.21	-0.90	-4.01

Note: Conditional estimates are descriptive predictions from difference-on-paired-mean models evaluated at selected paired means. Because the paired difference and paired mean are mathematically coupled and the compared methods have unequal marginal variances, these estimates should not be interpreted as disagreement at known levels of true adiposity or as independent evidence of an adiposity-level effect.

Supplementary Table S5. Absolute participant-level differences and complementary intraclass correlation coefficients.

Comparison	Median absolute difference, pp	IQR, pp	ICC(A,1), absolute agreement (95% CI)	ICC(C,1), consistency (95% CI)
Carter–Faulkner	4.33	3.64–5.06	0.693 (-0.046 to 0.917)	0.953 (0.913 to 0.975)
Carter–Omron	11.30	6.35–16.20	0.307 (-0.094 to 0.658)	0.659 (0.440 to 0.804)
Carter–InBody	5.71	1.91–10.95	0.506 (-0.023 to 0.771)	0.677 (0.466 to 0.815)
Faulkner–Omron	6.02	2.65–13.18	0.394 (-0.051 to 0.682)	0.567 (0.314 to 0.745)
Faulkner–InBody	4.58	2.37–7.51	0.617 (0.385 to 0.776)	0.626 (0.394 to 0.783)
Omron–InBody	5.25	2.98–7.45	0.773 (-0.037 to 0.933)	0.919 (0.853 to 0.957)

Note: ICC estimates are complementary measures and should not be interpreted as substitutes for Bland–Altman agreement. High consistency can coexist with systematic mean differences or wide limits of agreement. CI, confidence interval; ICC, intraclass correlation coefficient; IQR, interquartile range; pp, percentage points.

Supplementary Table S6. Exploratory sex-stratified and sex- and age-adjusted analyses.

Panel A. Sex-stratified mean differences and classical limits of agreement.

Comparison	Sex	n	Mean difference, pp	Bootstrap 95% CI	Classical 95% LoA, pp
Carter–Faulkner	Female	19	-3.21	-3.83 to -2.50	-6.13 to -0.29
Carter–Faulkner	Male	21	-5.01	-5.34 to -4.69	-6.53 to -3.49
Carter–Omron	Female	19	-15.83	-17.12 to -14.47	-21.87 to -9.78
Carter–Omron	Male	21	-6.59	-8.45 to -4.92	-14.97 to 1.80
Carter–InBody	Female	19	-8.62	-10.53 to -6.65	-17.28 to 0.04
Carter–InBody	Male	21	-2.94	-5.05 to -1.01	-12.60 to 6.71
Faulkner–Omron	Female	19	-12.62	-14.03 to -11.00	-19.21 to -6.03
Faulkner–Omron	Male	21	-1.57	-3.33 to 0.07	-9.46 to 6.31
Faulkner–InBody	Female	19	-5.41	-7.17 to -3.42	-13.77 to 2.95
Faulkner–InBody	Male	21	2.07	-0.04 to 3.90	-7.12 to 11.26
Omron–InBody	Female	19	7.21	5.99 to 8.52	1.57 to 12.85
Omron–InBody	Male	21	3.64	2.36 to 4.96	-2.45 to 9.73

Panel B. Sex- and age-adjusted sensitivity analyses of difference-on-paired-mean slopes.

Comparison	Crude slope	95% CI	Adjusted slope	95% CI	Absolute attenuation
Carter–Omron	-0.662	-0.866 to -0.459	-0.359	-0.579 to -0.139	45.8%
Carter–InBody	-0.582	-0.798 to -0.367	-0.471	-0.729 to -0.213	19.1%
Faulkner–Omron	-0.721	-0.973 to -0.470	-0.326	-0.544 to -0.108	54.8%
Faulkner–InBody	-0.622	-0.862 to -0.382	-0.432	-0.678 to -0.186	30.5%

Note: Sex-stratified and sex- and age-adjusted analyses were exploratory. The modest sex-specific sample sizes, differences in body-fat distributions by sex, and mathematical coupling inherent in difference-on-paired-mean regressions preclude interpretation of these results as evidence of sex-specific method effects or independent adiposity-level effects. LoA, limits of agreement; pp, percentage points.

Supplementary Table S7. Deming regression sensitivity analyses across assumed error-variance ratios.

Comparison	Assumed λ	Intercept (B on A)	Slope (B on A)
Carter–Faulkner	0.25	4.067	1.006
Carter–Faulkner	0.50	4.278	0.992
Carter–Faulkner	1.00	4.538	0.976
Carter–Faulkner	2.00	4.789	0.961
Carter–Faulkner	4.00	4.982	0.948
Carter–Omron	0.25	-9.726	2.291
Carter–Omron	0.50	-8.821	2.234
Carter–Omron	1.00	-7.252	2.136
Carter–Omron	2.00	-4.894	1.989
Carter–Omron	4.00	-2.129	1.817
Carter–InBody	0.25	-12.443	2.127
Carter–InBody	0.50	-11.415	2.063
Carter–InBody	1.00	-9.673	1.955
Carter–InBody	2.00	-7.177	1.799
Carter–InBody	4.00	-4.465	1.630
Faulkner–Omron	0.25	-26.767	2.663
Faulkner–Omron	0.50	-24.925	2.572
Faulkner–Omron	1.00	-21.637	2.409
Faulkner–Omron	2.00	-16.507	2.155
Faulkner–Omron	4.00	-10.395	1.852
Faulkner–InBody	0.25	-25.099	2.316
Faulkner–InBody	0.50	-23.451	2.235
Faulkner–InBody	1.00	-20.603	2.094
Faulkner–InBody	2.00	-16.419	1.886
Faulkner–InBody	4.00	-11.817	1.659
Omron–InBody	0.25	-4.319	0.962
Omron–InBody	0.50	-3.714	0.940
Omron–InBody	1.00	-3.000	0.913
Omron–InBody	2.00	-2.348	0.889
Omron–InBody	4.00	-1.875	0.872

Note: Deming regression estimates depend on the assumed ratio of method-specific measurement-error variances (λ). Because replicate measurements and independent estimates of measurement-error variance were unavailable, λ could not be empirically identified. These results are assumption-based sensitivity analyses and should not be interpreted as resolving proportional measurement differences independently of measurement error.

Supplementary Table S8. Influence diagnostics and leave-one-participant-out robustness analyses.

Comparison	Cook > 4/n, n (IDs)	Maximum Cook's distance	Max ∣mean-difference shift∣, pp	Max ∣lower LoA shift∣, pp	Max ∣upper LoA shift∣, pp
Carter–Faulkner	2 (13, 20)	0.879	0.135	0.366	0.635
Carter–Omron	1 (20)	0.145	0.273	0.351	0.628
Carter–InBody	1 (20)	0.186	0.277	0.622	0.711
Faulkner–Omron	1 (13)	0.103	0.288	0.457	0.620
Faulkner–InBody	1 (13)	0.118	0.286	0.553	0.707
Omron–InBody	2 (12, 16)	0.119	0.240	0.421	0.846

Note: Participant IDs are anonymized analytical identifiers. Cook's distance > 4/n was used only as a screening flag. No observation was excluded on this basis. Leave-one-participant-out shifts are absolute changes from the full-sample estimate. Several observations exceeded the conventional screening threshold, but no single exclusion materially altered the overall interpretation. LoA, limits of agreement; pp, percentage points.

Supplementary Table S9. Precision of limits of agreement and illustrative sample-size requirements for future studies.

Panel A. Precision of classical limits of agreement in the current sample.

Comparison	SD of differences, pp	Classical 95% LoA, pp	Bootstrap 95% CI lower LoA	Bootstrap 95% CI upper LoA	Approx. analytic 95% CI half-width, pp
Carter–Faulkner	1.47	-7.04 to -1.27	-7.71 to -6.26	-2.48 to -0.07	0.81
Carter–Omron	5.97	-22.67 to 0.72	-24.50 to -20.19	-2.53 to 3.06	3.29
Carter–InBody	5.45	-16.32 to 5.04	-18.38 to -13.66	2.57 to 7.02	3.00
Faulkner–Omron	6.69	-19.93 to 6.29	-22.03 to -17.01	3.11 to 8.63	3.69
Faulkner–InBody	5.83	-12.91 to 9.94	-14.96 to -10.13	7.22 to 12.01	3.21
Omron–InBody	3.47	-1.46 to 12.13	-2.97 to 0.27	10.14 to 14.04	1.91

Panel B. Illustrative sample-size requirements for specified 95% confidence-interval half-widths around the limits of agreement.

Comparison	±1.0 pp	±1.5 pp	±2.0 pp	±2.5 pp	±3.0 pp
Carter–Faulkner	28	14	10	7	6
Carter–Omron	403	181	103	68	48
Carter–InBody	337	152	87	57	41
Faulkner–Omron	505	227	129	84	59
Faulkner–InBody	385	173	99	65	46
Omron–InBody	139	64	37	25	19

Note: Panel A: Bootstrap confidence intervals for individual LoA were obtained using participant-level percentile resampling with 10,000 replicates. The approximate analytical 95% CI half-width is a separate precision descriptor and should not be interpreted as the half-width of the asymmetric bootstrap intervals or as a retrospective power measure. Panel B: Approximate required sample sizes were calculated from the observed SD of paired differences for planning future method-comparison studies. These values are illustrative and should not be interpreted as retrospective power calculations or normative sample-size requirements.
